# Exploring the role of unnatural amino acids in antimicrobial peptides

**DOI:** 10.1038/s41598-018-27231-5

**Published:** 2018-06-11

**Authors:** Rosario Oliva, Marco Chino, Katia Pane, Valeria Pistorio, Augusta De Santis, Elio Pizzo, Gerardino D’Errico, Vincenzo Pavone, Angela Lombardi, Pompea Del Vecchio, Eugenio Notomista, Flavia Nastri, Luigi Petraccone

**Affiliations:** 1 Department of Chemical Sciences, University of Naples “Federico II”, via Cintia, I-80126 Naples, Italy; 2 Department of Biology, University of Naples “Federico II”, Via Cintia, I-80126 Naples, Italy; 3 Department of Molecular Medicine and Medical Biotechnologies, University of Naples “Federico II”, Via Pansini, 5, I-80131 Naples, Italy

## Abstract

Cationic antimicrobial peptides (CAMPs) are a promising alternative to treat multidrug-resistant bacteria, which have developed resistance to all the commonly used antimicrobial, and therefore represent a serious threat to human health. One of the major drawbacks of CAMPs is their sensitivity to proteases, which drastically limits their half-life. Here we describe the design and synthesis of three nine-residue CAMPs, which showed high stability in serum and broad spectrum antimicrobial activity. As for all peptides a very low selectivity between bacterial and eukaryotic cells was observed, we performed a detailed biophysical characterization of the interaction of one of these peptides with liposomes mimicking bacterial and eukaryotic membranes. Our results show a surface binding on the DPPC/DPPG vesicles, coupled with lipid domain formation, and, above a threshold concentration, a deep insertion into the bilayer hydrophobic core. On the contrary, mainly surface binding of the peptide on the DPPC bilayer was observed. These observed differences in the peptide interaction with the two model membranes suggest a divergence in the mechanisms responsible for the antimicrobial activity and for the observed high toxicity toward mammalian cell lines. These results could represent an important contribution to unravel some open and unresolved issues in the development of synthetic CAMPs.

## Introduction

The wide range of chemical diversity achievable with peptides make them good candidates as therapeutic agents^1^. Indeed, several peptide-based drugs have reached the market and are now in daily use^2^. This field has strongly expanded in the last two decades, thanks to the increasing availability of design strategies and versatile synthetic approaches to develop peptide sequences. Taking inspiration from natural occurring antimicrobial peptides, many efforts have been devoted to the development of peptides as alternative antimicrobial agents respect to conventional antibiotics^3,4^. These molecules could represent a solution to the urgent problem of treating infectious diseases caused by multidrug-resistant bacteria^5^.

Antimicrobial peptides (AMPs) are involved in the defence mechanisms found in every forms of life^5,6^. They are a very heterogeneous group of antimicrobials with different lengths (usually from 12–13 to more than 70–80 residues) and structures. Their mechanism of action^7,8^ is also very variable, ranging from the alteration of membranes, to targeting specific metabolic pathways or cellular components. The majority of AMPs targeting membrane bilayers are CAMPs^5,7,8^. They contain arginine or lysine residues (generally from 2 to 9), which account for the positive net charge of CAMPs. Further, they share a common amphiphilic structure, in which cationic and hydrophobic residues are clustered in different spatial regions. This organization, observed within a variety of secondary structures, accounts for their mechanism of interaction with membranes^9^. First, their net positive charge allows their accumulation at the surface of bacterial cells, which are generally rich of anionic groups. In particular, anionic lipids like lipopolysaccharides (LPS) characterize the outer membrane of Gram-negative strains, whether phosphatidylglycerol and cardiolipin are present in the (inner)membrane of the majority of bacteria^10–12^. Subsequently, the hydrophobic residues play key roles for the insertion of the CAMP into the membrane^13^. Interactions with membrane can cause different damaging actions, such as membrane-penetration, pore-formation and membrane disruption^5,14–16^, which in turn causes the inhibition of bacterial growth or death.

Several models have been proposed for describing the complex mechanisms of action, spanning from the “carpet model”, the “barrel stave model” and the “toroidal pore model”^7,12,17,18^. According to the carpet model, CAMPs would lay on the surface of the membrane forming a sort of carpet, as their concentration increases. Cell damage and death would be the result of phase separation – i.e. segregation of anionic and zwitterionic lipids – membrane tinning, destabilization and, lastly, lysis. According to the barrel stave model, CAMPs would adopt an orientation perpendicular to the surface of membrane creating a stable pore lined only by peptide molecules. Currently, it is believed that this model is correct only for few CAMPs^19^. According to the toroidal pore model, CAMPs assume an orientation perpendicular to the membrane surface, however, due to a strong interaction between peptides and phospholipid heads, membrane surface would bend thus leading to the formation of a pore lined by both peptides and phospholipid heads. This model does not require direct peptide/peptide contacts, moreover, it has been demonstrated that a toroidal pore can be stabilized even by a single peptide molecule^20^. Differently from the barrel stave pores, the toroidal pores would be dynamic structures which continuously form and disassemble. According to some authors, the carpet and the toroidal pore models are not mutually exclusive and could be both true depending on peptide/lipid ratio, membrane type and experimental conditions^17^.

A key issue to be addressed for the development of synthetic peptides as antimicrobial agents is to develop sequences that display both resistance to proteolysis and selective toxicity^21^. Indeed, the therapeutic applications of CAMPs are limited by their sensitivity to proteases, which drastically reduces their half-life. Higher eukaryotes have adopted several strategies to overcome this drawback. For example, CAMPs can be produced in a tightly regulated manner directly at the site of the infection. Moreover, often, CAMPs are secreted in complex mixtures^22^ thus reducing the risk that a microbial protease could degrade all the components. An even more surprising strategy is the production of proteins, not necessarily related to host defence, containing CAMP-like sequences in their primary structure^23–27^. These “cryptic” CAMPs can be released by the action of host and/or bacterial proteases so that if a bacterium secretes a protease to inactivate CAMPs at the same time it will cause the release of further peptides^23,28^. From the pharmacological point of view, to improve their suitability as drugs, several researches have developed CAMPs with modifications that reduce sensitivity to proteases, like blocking N- and C-termini, introducing D-amino acids at key positions etc., or peptidomimetics inspired to CAMPs but not hydrolysable^29,30^. However, also an excessive resistance to hydrolysis could be unfavorable from a pharmacological point of view, causing bioaccumulation and increased toxicity.

Further, to function as effective drugs, antimicrobial synthetic peptides should act selectively on the bacterial cells and be inactive on host cells. The different composition between prokaryotic and eukaryotic cell membranes is one of the main target to address for designing selective AMPs. In eukaryotic membranes the anionic lipids (e.g. phosphatidylserine) are only located in the inner leaflet, whereas, the outer leaflet is mainly composed of lipids with no net charge. Thus, cationic AMPs have in principle high affinity for the negatively charged bacterial membranes respect to those of plants and animals^6^. However, this aspect is not sufficient to avoid their toxicity toward mammalian cells^31^.

**Figure 1:**
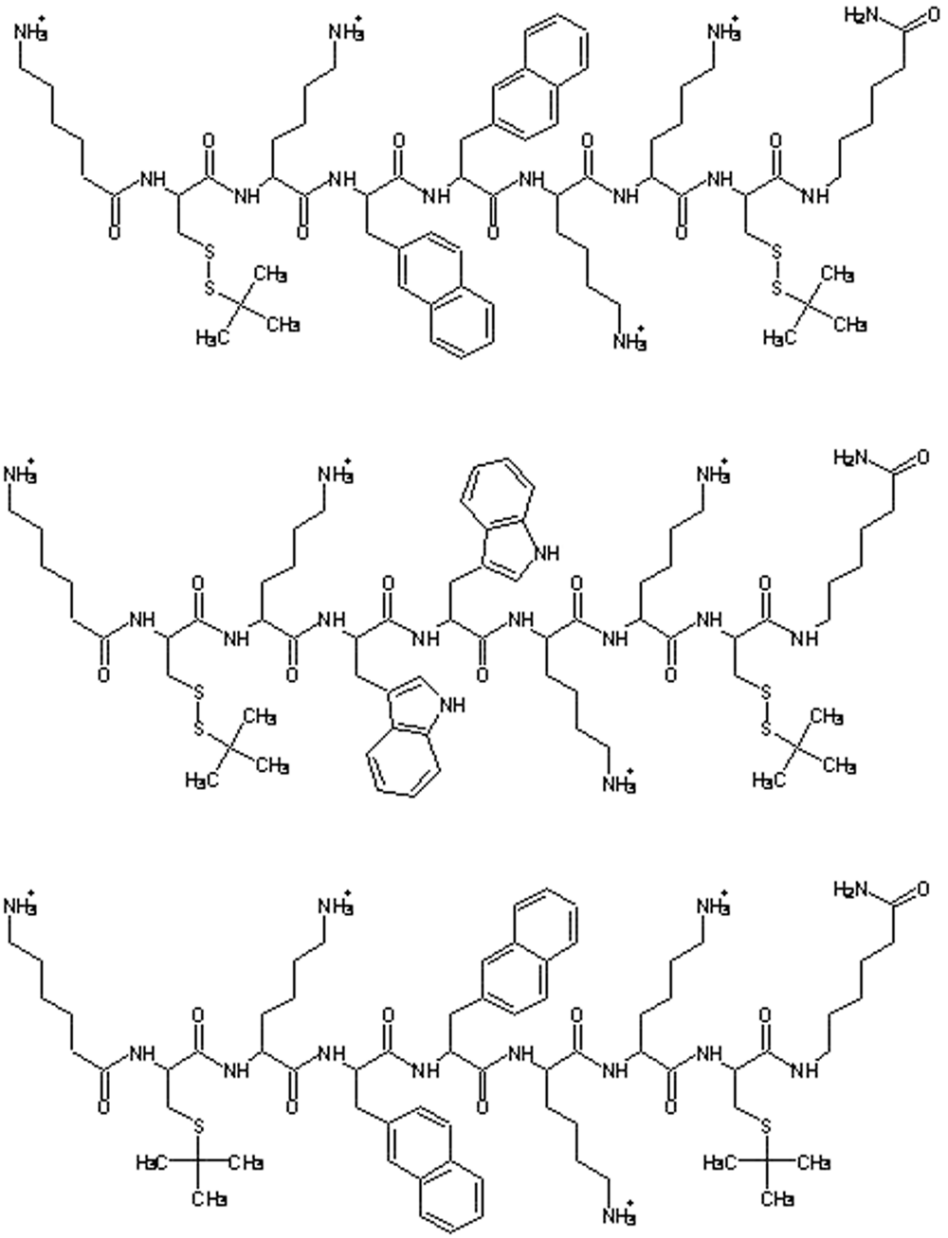
Structures of peptides P9Nal(SS), P9Trp(SS), and P9Nal(SR).

CAMPs therapeutic potential is therefore still limited, and many efforts have been devoted to analyze the molecular basis required to achieve stability and cell selectivity. In this respect, one facet that is still poorly investigated is the interaction of CAMPs with eukaryotic membranes^32^. Here we report the antimicrobial activity of three synthetic nona-peptides, containing cationic and hydrophobic residues, as CAMP mimics. Each peptide includes three lysine residues, two aromatic residues (either tryptophan or 2-naphthyl-L-alanine), two cysteine derivatives (a thioether or a mixed disulfide) and N- and C-terminal 6-aminohexanoic acid residues introduced to protect peptides from the action of exopeptidases (see Fig. 1).

All the peptides display high activity toward a broad spectrum of pathogens. Further, insertion of un-natural amino acids in their sequences was effective in increasing their stability. As their antimicrobial activity strongly correlate with a concomitant undesired cytotoxicity, we performed a detailed biophysical characterization of the interaction of one of these peptides with liposomes of different lipid composition mimicking bacterial and eukaryotic membranes. The peptide appeared to differently interact with the two model bio-membranes, thus suggesting a divergence in the mechanisms responsible for the antimicrobial activity and for the observed high toxicity toward mammalian cell lines. These results could represent an important contribution to unravel some open and unresolved issues in the development of synthetic CAMPs as effective drugs.

## Materials and Methods

### Materials

Fmoc protected amino acid and coupling reagents were purchased from NovaBiochem and used without further purification. H-PAL Chemmatrix resin was from Sigma Aldrich. Peptide synthesis and purification solvents were from Romil (Biopure grade). Piperidine and N,N’-diisopropyl ethylamine were from NovaBiochem. The fluorescent probe DPH (1,6-Diphenyl-1,3,5-hexatriene) and acrylamide solution (40% w/v) were obtained from Sigma Aldrich Chemical. The lipids 1,2-dipalmitoyl-*sn*-glycero-3-phosphocholine (DPPC), 1,2-dipalmitoyl-sn-glycero-3-phospho-1′-rac-glycerol (DPPG) and 1-palmitoyl-2-oleoyl-sn-glycero-3-phospho-1′-rac-glycerol (POPG) were purchased from Avanti Polar Lipids Inc. (Alabaster, AL, USA) and used without further purifications. Spin-labeled phosphatidylcholines (1-palmitoyl-2-stearoyl-(n-doxyl)-sn-glycero-3-phosphocholine, *n*-PCSL) with the nitroxide group in the positions 5, 14 of the acyl chain for EPR experiments were also obtained from Avanti Polar Lipids. The chemical structures of all the lipids used are shown in Fig. S1. Deionized water was used for the phosphate buffer and all sample preparations.

### Peptide Synthesis

Peptides were synthesized by standard Fastmoc protocols on an ABI 433 A automatic peptide synthesizer^33^. Dry H-PAL Chemmatrix resin (0.31 mmol g^−1^ substitution) was weighed to get a 0.25 mmol scale, and manually swelled in N-Methyl-2-pyrrolidone (NMP). Double coupling steps were performed for the two bulky aromatic residues. After the final deprotection step, the resin was washed with 2-propanol and with methanol and then dried. Cleavage and deprotection was performed with 25 mL of a 95:2.5:2.5 trifluoroacetic acid (TFA): tri-isopropyl silane (TIS):H_2_O solution. Cleavage reaction was performed for two hours, the first at 0 °C and the second at room temperature under gentle magnetic stirring. The exhaust resin was washed three times with fresh TFA and then discarded by filtration. The combined filtrates were concentrated in vacuo and the crude peptide precipitated with five volumes of diethyl ether. Crude peptide was washed three times with diethyl ether to remove the scavenged groups and then dried to get a 44% final yield.

### Peptide Purification

Crude peptides were purified by preparative HPLC on a Shimadzu LC-8A system. A Vydac C18 column (22 mm × 250 mm; 10 μm) was used, eluted with a H_2_O 0.1% TFA, (eluent A) and CH_3_CN 0.1% TFA (eluent B) linear gradient at 22 mL min^−1^ flow rate. Fractions were separated according to their absorbance at 210 nm by an online UV detector (Shimadzu). Peptide fractions were then checked for their purity by analytical LCMS. Peptide identities were confirmed by LCMS analysis on a Shimadzu 20ADxr coupled with a Shimadzu ESI-IT-TOF mass detector (probe voltage 4.5 kV; probe temperature 25 °C; desolvation temperature 250 °C; detector voltage 1.5 kV). All analyses were performed with a Vydac C18 column (2.1 mm × 100 mm; 5 μm), eluted with a H_2_O 0.05% TFA, (eluent A) and CH_3_CN 0.05% TFA (eluent B) linear gradient, from 5% to 95% (solvent B), over 30 minutes, at 0.2 ml min^−1^ flow rate. Pure fractions were pooled and lyophilized as TFA salts (purity >95%). Peptide samples for biological assays were further subjected to counter ion exchange on a DEAE-Sephadex weak acidic resin. Briefly, peptide solutions in water were loaded on a 0.5 mL syringe column, and then eluted by decreasing the pH with an acetate buffer at pH 4.3 to obtain the desired peptide acetate salt. Further, the relative hydrophobicities of P9 peptides were evaluated through their retention in reverse phase HPLC.

### Biological Assays

#### Antimicrobial activity assay (standard condition)

The antimicrobial activity of the three designed peptides was tested against *Escherichia coli* (ATCC*®* 25922*™*), *Pseudomonas aeruginosa* (ATCC*®* 27853*™*), *Pseudomonas aeruginosa* PAO1 wild type strain, *Bacillus subtilis* subsp. *spizizenii* (ATCC*®* 6633*™*), *Staphylococcus aureus* (ATCC*®* 6538 P*™*) and *Staphylococcus aureus* MRSA WKZ-2 (kindly provided by collection of Prof. Edwin Veldhuizen, University of Utrecht, Holland). MIC assays were performed by broth microdilution method as described elsewhere^34^ using Nutrient Broth (Becton Dickinson Difco, Franklin Lakes, NJ) as bacterial growth medium. Recombinant (P)GKY20 peptide^35^ was used as control antimicrobial peptide. MIC values were measured as the lowest concentration at which no visible growth was observed at the end of the incubation time. Experiments were performed three times for each peptide.

#### Antimicrobial activity of peptide pre-incubated in 10% serum

In addition to the antimicrobial activity assays performed according to the standard protocol, MIC values of the three designed peptides were determined against *Escherichia coli* ATCC 25922, also upon peptides incubation in 10% serum (fetal bovine serum, Microgem Lab, Cat. S1860, Italy) for 1 hour or 16 hours at 37 °C (water bath). Following the incubation time, MIC assays were carried out as previously described. Recombinant peptides (P)GKY20 and ApoE(133–150) and synthetic peptide Ac-ApoE(133–150)-NH_2_ with acetylated N-terminus and amidated C-terminus^25,35^ were treated as the three designed peptides and used as control antimicrobial peptides. Experiments were performed five times for each peptide.

#### Cytotoxicity Assay

Cytotoxic effects of the three peptides on human epithelial colorectal adenocarcinoma cells (CaCo-2) and on aneuploid immortal keratinocyte cells line from adult human skin (HaCaT) were assessed by performing the (3-(4,5-dimethylthiazol-2-yl)−2,5 diphenyltetrazolium bromide (MTT) reduction inhibition assay^36^. Both human cell lines were obtained from the American Type Culture Collection (ATCC, Manassas, VA, USA) and grown at 37 °C in a humidified incubator containing 5% CO_2_ in Dulbecco’s modified Eagle’s medium (DMEM) supplemented with 10% fetal bovine serum, 4 mM glutamine, 400 units/mL penicillin, and 0.1 mg mL^−1^ streptomycin. Cells were plated on 96-well plates at a density of 5 × 10^3^ per well in 100 μL of medium and incubated at 37 °C with 5% CO_2_. Medium was then replaced with 100 μl of fresh media containing peptide solution to a final concentration ranging from 0.1–10 μM/well. After a time of incubation ranging from 18 to 48 hours at 37 °C, the peptide-containing medium was removed, and 10 μL of a 5 mg mL^−1^ MTT stock solution in DMEM without red phenol, corresponding to a final concentration of 0.5 mg L^−1^ in DMEM (final volume of 100 μL), was added to the cells. After 4 h of incubation, the MTT solution was removed and the MTT formazan salts were dissolved in 100 μL of 0.1 N HCl in anhydrous isopropanol. Cell survival was reported as the relative absorbance, with respect to control, of blue formazan measured at 570 nm with a Synergy Multi Plate Reader. Cytotoxicity experiments were performed at least three times independently. Standard deviations were always <10% for each experiment.

### Liposome Preparation

An appropriate amount of lipids was weighted in a glass vial and dissolved in a chloroform/methanol mixture (2/1 v/v). A thin film was produced by evaporating the organic solvent with dry nitrogen gas. The sample was placed in a vacuum for at least 3 h. The dried lipids were then hydrated, in the liquid-crystalline phase at the temperature of 50 °C, with an appropriate amount of 20 mM phosphate buffer pH 7.4, and vigorously vortexed obtaining a suspension of multilamellar vesicles (MLVs). Small unilamellar vesicles (SUVs) were obtained using Sonics VCX130 tip sonicator until the suspension became transparent (about 20 min, at room temperature). Dynamic light scattering (DLS) measurements were used to check the size of lipid vesicles after sonication. The obtained average of the hydrodynamic radii of SUVs (∼90 nm) are consistent with the formation of unilamellar vesicles. SUVs containing the fluorescent probe DPH (1,6-Diphenyl-1,3,5-hexatriene) were obtained by adding to the lipids dissolved in the organic mixture a definite amount of a solution of DPH in chloroform at the lipids/DPH mole ratio of 200. Liposomes with different composition were prepared: i) DPPC, as a model of eukaryotic membrane; ii) DPPC/DPPG (8/2 mol/mol) and DPPC/POPG (8/2 mol/mol) as models of bacterial membrane. Samples of liposomes in the presence of peptide were prepared by mixing appropriate volumes of peptide in solution and liposomes suspension to get the desired lipid-to-peptide (L/P) ratio.

### Circular Dichroism

Circular dichroism (CD) spectra of peptide P9Nal(SS) were recorded using a JASCO J-715 spectropolarimeter (Jasco Corporation, Tokyo, Japan) in a 0.1 cm path length quartz cuvette as an average of 3 scans. The instrument parameters were set as follows: scan speed of 20 nm/min, 4 s response time and 2 nm bandwidth. The temperature was set to 25 °C. Peptide samples were prepared in 20 mM phosphate buffer pH 7.4 at the concentration of 35 μM in absence and in the presence of SUVs at total lipid concentration of 0.35 mM, 1.75 mM and 3.5 mM. For each sample, a background blank (buffer or lipid suspension alone) was subtracted. The peptide concentration was determined by means of UV-Vis measurements using a molar extinction coefficient of ε_277nm_ = (9109 ± 73) M^−1^cm^−1^ (Fig. S2).

### Differential Scanning Calorimetry

DSC measurements were performed using a nano-DSC from TA instruments (New Castle, DE, USA). MLVs were used for all DSC experiments since they provide the better resolution of the peak^37^. A volume of 300 μL of 0.5 mM vesicles suspension (DPPC, DPPC/DPPG or DPPC/POPG) in the absence or in the presence of peptide was placed in the calorimetry vessel, and successive heating and cooling scans were performed at 1 °C/min over the temperature range of 20–55 °C.

The excess heat capacity function (<ΔC_p_>) was obtained after baseline subtraction. A buffer-buffer scan was subtracted from the sample scan^38^. The samples composed by lipid suspension and peptide were prepared just before the DSC experiments, by adding the appropriate amount of peptide to the lipid suspension and waiting at least 30 min to ensure that the equilibrium has been reached. The results presented here all refer to the second heating scan. The obtained data were analyzed by means of NanoAnalyze software supplied with the instrument and plotted using the Origin software package (OriginLab, Northampton, MA, USA).

### Steady-state Fluorescence

All the fluorescence experiments were performed using a Fluoromax-4 spectrofluorometer (Horiba, Edison, NJ, USA) operating in the steady-state mode at the temperature of 25 °C.

*Fluorescence anisotropy*. Fluorescence anisotropy measurements were carried out for the probe DPH embedded into SUVs at total lipid concentration of 150 µM. The excitation wavelength was set to 355 nm and the emission was monitored at 427 nm. The slits were set to 4 nm for both the excitation and emission monochromators. The experiments were performed using a 1 cm path length quartz cuvette. Fluorescence anisotropies (r) were determined according to the relation:

1 $$r=\frac{{I}_{VV}-G{I}_{VH}}{{I}_{VV}+2G{I}_{VH}}$$

where I_VV_ is the fluorescence intensity obtained by setting both the excitation and emission polarizers vertically, I_VH_ is the fluorescence intensity obtained by setting the excitation polarizer vertically and the emission polarizer horizontally and G is the instrument-specific correction factor^39^.

#### Fluorescence Resonance Energy Transfer

In the FRET experiments, the P9Nal(SS) peptide in solution was excited in the presence of SUVs labeled with DPH. The excitation wavelength was set to 277 nm and emission spectra were recorded in the range 300–510 nm, using a 1 cm path length quartz cuvette. The slits for the excitation and emission monochromators were set to 3 nm and 6 nm, respectively. As references the spectra of peptide in buffer or in the presence of vesicles without DPH were also collected. From each sample, a blank (vesicles in the absence of the peptide or buffer solution) measurement was subtracted. The concentration of peptide was fixed at 5 μM and spectra at L/P ratio of 50 were recorded.

#### Fluorescence quenching

For fluorescence quenching experiments, a 7 μM solution of peptide, in the absence or in the presence of SUVs at lipid-to-peptide mole ratio (L/P) of 100, was placed in a quartz cuvette with a path length of 1 cm and titrated with acrylamide solution (40% w/v). The titrations were performed at fixed peptide concentration in the absence and presence of acrylamide concentrations up to ~50 mM. The excitation wavelength was set to 277 nm, and the emission spectra were collected from 290 nm to 500 nm. The obtained data were analyzed using the Stern-Volmer equation:

2 $${{\rm{F}}}_{0}/{\rm{F}}=1+{{\rm{K}}}_{{\rm{SV}}}[{\rm{Q}}]$$

where F_0_ is the fluorescence intensity in the absence of the quencher, F is the fluorescence intensity at each step of titration in the presence of the quencher, [Q] is the concentration of acrylamide^39^. Due to acrylamide absorption at the excitation wavelength, the fluorescence intensities were corrected using the formula ${F}_{obs}={F}_{corr}\cdot {10}^{\frac{Aex\ast d}{2}}$ where F_obs_ is the observed intensity, F_corr_ is the corrected intensity, A_ex_ is the absorbance of acrylamide at the excitation wavelength and d is the cuvette path length.

### Electron Paramagnetic Resonance

EPR experiments were performed on MLVs prepared as described above. Spin-labeled phosphatidylcholines (5-PCSL or 14-PCSL) were added to the lipid mixture (1% mol on the total lipid) by mixing appropriate amounts of a spin-label solution in ethanol (1 mg/mL) with the lipid organic mixture. MLV suspensions were transferred into 25 μL glass capillaries, and immediately sealed. Samples containing P9Nal(SS) were prepared by the same procedure, adding appropriate amounts of a stock solution of the peptide dissolved in 10 mM phosphate buffer at pH = 7.4, in order to obtain L/P ratio of 100, 50, 30, 25, 10.

EPR spectra were recorded with a 9 GHz Bruker Elexys E-500 spectrometer (Bruker, Rheinstetten, Germany). The capillaries containing MLVs suspensions to be investigated were placed in a standard 4 mm quartz sample tube containing light silicone oil for thermal stability. All the measurements were performed at 25 °C. Spectra were recorded using the instrumental settings optimized in a previous work^40^. In order to quantitatively analyze the spectra, we evaluated the order parameter, S, and the isotropic hyperfine coupling constant, a′_N_. Briefly, these parameters were calculated according to the relations:

3 $${a^{\prime} }_{N}=\frac{1}{3}({T}_{\parallel }+2{T}_{\perp })$$

4 $$S=\frac{({T}_{\parallel }-{T}_{\perp })}{({T}_{zz}-{T}_{xx})}\,\frac{{a}_{N}}{{a}_{N}^{^{\prime} }}$$

where ${T}_{\parallel }$ and ${T}_{\perp }$ are two phenomenological hyperfine splitting parameters which can be determined from the positions of minima and maxima in the spectra. Reliable values of the magnetic field corresponding to minima and maxima were determined by using a MATLAB based routine^41^. *T*_xx_ and *T*_zz_ are the principal elements of the real hyperfine splitting tensor in the spin Hamiltonian of the spin-label, as determined from the corresponding single-crystal EPR spectrum (*T*_xx_ = 6.1 G and *T*_zz_ = 32.4 G). *a*_*N*_ is the isotropic hyperfine coupling constant for the spin-label in crystal state, given by:

5 $${a}_{N}=\frac{1}{3}({T}_{zz}+2{T}_{xx})$$

## Results

### Biological Study

#### Design

The three peptides used in this work were intended to mimic the activity of naturally occurring CAMPs. To this aim, peptide sequences containing an alternation of polar/positively charged and hydrophobic residues were designed. Further, un-natural amino acids were introduced to increase both hydrophobicity and stability to proteases (see Fig. 1). In details, the sequences incorporate three lysine, selected as cationic residues, and all contain 6-aminohexanoic acid (εAhx) at both N- and C-termini. The terminal εAhx residues were introduced for their known ability to protect peptides from the action of exopeptidases, and for their aliphatic tail, which contributes to the overall peptide hydrophobicity^42^. Further, their presence account for an additional positive charge at the free unprotected N-terminus. Hydrophobicity was further modulated by insertion of aromatic residues, such as 2-naphthyl-L-alanine (2-Nal) and tryptophan. These residues have been already demonstrated to increase the antibacterial activity in various peptide sequences^43,44^. Finally, two side chain protected Cys residues, as thioether (CysS-tBu) or disulfide (Cys-S-S-ter-butyl-thio), were inserted into the sequences named SR or SS, respectively. Several naturally occurring CAMPs are characterized by a different contents of Cys residues, either forming disulfide bridges as in defensin, or forming tioether-based intramolecular ring as in lantibiotics^4^. Recent studies demonstrated that upon disulfide reduction, several AMPs show potent activity against some bacterial strains^45^. Therefore, we tried to explore the eventual role of reduced and/or oxidized form of Cys-containing synthetic CAMPs, by comparing the activity of the different sequences.

#### Antimicrobial activity and serum stability

The antimicrobial activity of the three designed peptides was determined against Gram-negative and Gram-positive bacteria strains. MIC values (minimum inhibitory concentration leading to no visible bacterial growth) ranged from 10 to 1.25 µM against Gram-negative bacteria strains, and from 40 to 10 µM against Gram-positive bacteria strains. Further, the three peptides showed antibacterial activity similar to the control peptide (P)GKY20, the recombinant counterpart of GKY20, a “natural” cryptic antimicrobial peptide deriving from the C-terminal region of human thrombin^35,46^ (Table 1). Among the three designed peptides, P9Nal(SS) possesses broader spectrum antibacterial activity compared to the other peptides as highlighted by the lower MIC values (10–20 µM) obtained in the case of Gram-positive bacteria strains.

**Table 1:** Antibacterial activity of the peptides.

MIC (µM)^a^						
Peptide	Gram-negative			Gram-positive		
	*E. coli* ATCC25922	*P. aeruginosa* ATCC27853	PAO1 Wt	*B. spizizenii* ATCC6633	*S. aureus* MRSA	*S. aureus* ATCC6538P
(P)GKY20	10	20	20	20	80	5
P9Nal(SS)	10	2.5	10	10	20	10
P9Trp(SS)	10	2.5	10	40	40	20
P9Nal(SR)	10	1.25	10	40	40	40

^a^MIC is the minimum inhibitory concentration leading to no visible bacterial growth. Assays were carried out three times as independent experiments.

We next assessed whether serum proteases affected the antimicrobial potency of the peptides by measuring MIC values of peptides pre-incubated in 10% serum (Fetal Bovine Serum, FBS) for 1 hour or 16 hours. Two recombinant peptides with free termini, (P)GKY20 and ApoE(133–150)^25,35^, and a synthetic peptide with protected termini, Ac-ApoE(133–150)-NH_2_^25^, were included as controls. Similarly to (P)GKY20, ApoE(133–150) is a cryptic antimicrobial peptide which derives from the receptor binding region of human apolipoprotein E^25^.

Table 2 summarizes the effect of serum pre-incubation on peptide activity on *Escherichia coli* ATCC 25922. The three designed peptides and control peptide (P)GKY20 did not show any change in the MIC values after preincubation in 10% serum for 1 h, whereas, ApoE(133–150) completely lost antimicrobial activity and Ac-ApoE(133–150)-NH_2_ showed a four fold increase in the MIC value. In the case of the preincubation for 16 h, all control peptides and the designed peptide P9Nal(SR) completely lost their activity (MIC values > 80 µM), whereas, P9Nal(SS) and P9Trp(SS) showed fourfold and eightfold increase in the MIC values, respectively. These data clearly show that both 2-Nal and S- *tert*-butylthio L-cysteine residues contribute to the resistance to serum proteases.

**Table 2:** Antibacterial activity of the three designed peptides against *Escherichia coli* ATCC 25922 after preincubation in 10% serum.

MIC (µM)^a^					
	No preincubation	Preincubation for 1 h in 10% serum	Preincubation for 16 h in 10% serum	Fold change (1 h)^b^	Fold change (16 h)^c^
(P)GKY20	10	10	>80	1	>8
ApoE(133–150)	5	>80	>80	>16	>16
Ac-ApoE(133–150)-NH_2_	5	20	>80	4	>16
P9Nal(SS)	10	10	40	1	4
P9Trp(SS)	10	10	80	1	8
P9Nal(SR)	10	10	>80	1	>8

^a^MIC is the minimum inhibitory concentration leading to no visible bacterial growth. Data reported are based on set of five replicates. Peptides were assayed before or after incubation for 1 hour or 16 hours in the presence of 10% serum (FBS) at 37 °C.

^b^and ^c^the fold change in antimicrobial activity are calculated as ratio between peptide MIC value upon incubation for 1 h (b) and 16 h (c) in presence of serum 10% at 37 °C and MIC values determined for the peptides not incubated in serum.

#### Toxicity for eukaryotic cell lines

**Figure 2:**
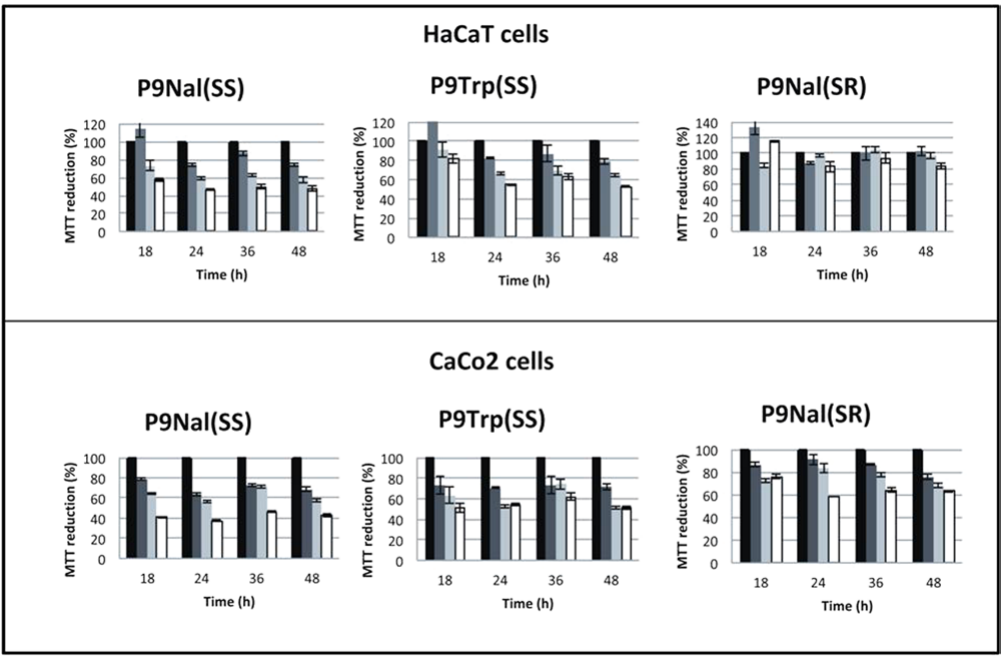
Toxicity MTT assay of the P9Nal(SS), P9Trp(SS) and P9Nal(SR) on human intestinal CaCo-2 cells and human keratinocytes cells HaCaT.

To assess the potential toxicity of the three designed peptides, the effects on human intestinal CaCo-2 cells and human keratinocytes cells HaCaT were evaluated by the MTT reduction assay at four different times of incubation (18, 24, 36 and 48 hours). The results are collected in Fig. 2 and showed that both P9Nal(SS) and P9Trp(SS) exert significant dose dependent toxic effects on both cell lines, whereas P9Nal(SR) seems to have no evident toxic effects on HaCaT cells when it was assayed at low doses (0.1 or 1 μM) and its effect is slightly higher on CaCo2 cells when it was assayed at the same doses.

### Biophysical Study

In order to obtain detailed information on the peptide-membrane interaction mechanism, we chose P9Nal(SS) for further biophysical studies as this peptide, among the three peptides, possesses broader spectrum antibacterial activity together with the higher stability in serum. Particularly, we characterized the interaction of P9Nal(SS) with DPPC and DPPC/DPPG liposomes mimicking the eukaryotic and bacterial membranes, respectively.

#### Circular Dichroism

**Figure 3:**
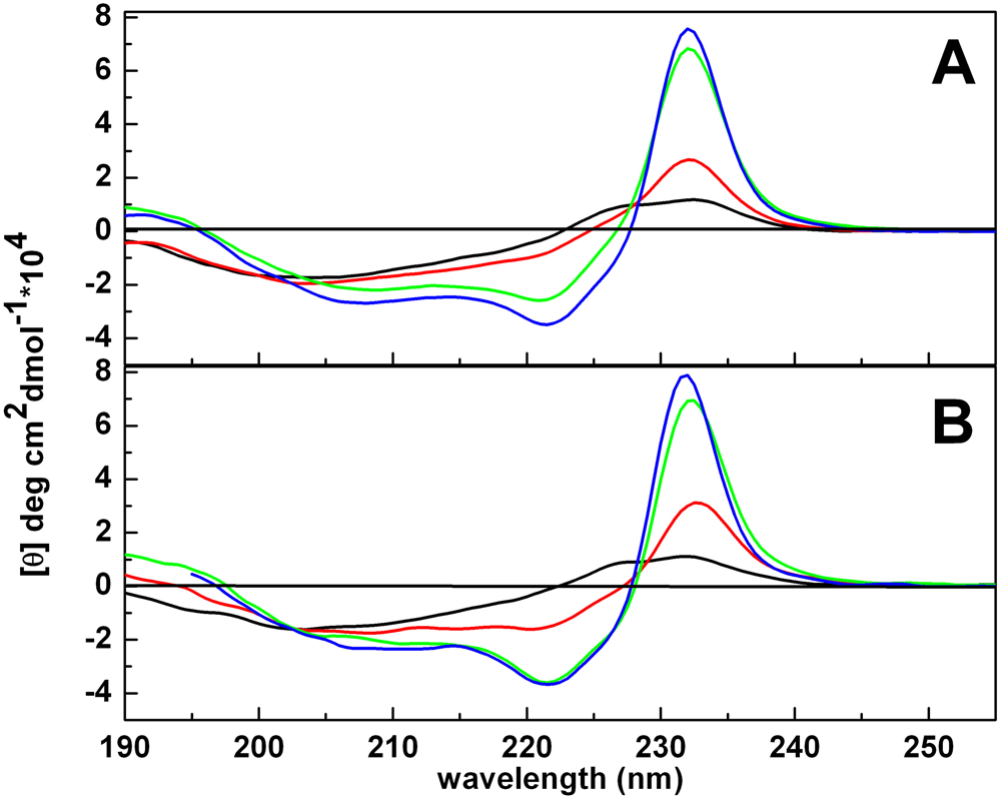
CD spectra of the peptide P9Nal(SS) in buffer (black line) and in the presence of DPPC (**A**) and DPPC/DPPG (**B**) SUVs at lipid-to-peptide ratio of 10 (red line), 50 (green line) and 100 (blue line).

Far-UV CD spectroscopy was used to analyze the conformational behaviors of the peptide P9Nal(SS). Figure 3 shows the CD spectra of the peptide in buffer solution and in the presence of small unilamellar vesicles (SUVs) of DPPC and DPPC/DPPG lipids, at L/P ratio ranging from 10 to 100. The CD spectrum of P9Nal(SS) in buffer solution is characterized by a positive band around 230 nm, and a broad negative band around 200 nm suggesting that the peptide assumes a disordered conformation in solution. By increasing the lipid concentration, for both DPPC and DPPC/DPPG vesicles, the intensity of the positive band increases and two minima at about 220 nm and 207 nm appear. In addition, at L/P ratio of 100 and 50, a positive band rises around 195 nm. The positive band of the peptide in buffer at about 230 nm is due to the contribution of the aromatic 2-Nal side chains as confirmed by the presence of this band in both P9Nal(SS) and P9Nal(SR) but not in the P9Trp(SS) peptide (Fig. S3).

The further strong increase of this positive band upon lipid addition to P9Nal(SS) could be due to an exciton effect, since the two 2-Nal residues are very close to each other and the interaction between the excited states contribute to the CD spectrum^47,48^. The exciton effect leads to the splitting of the excited state in two components opposite in signs. In our case, a positive band at 230 nm and a negative one at around 220 (positive couplet) were observed. Thus, upon the interaction with lipid vesicles, the peptide adopts an helical conformation where the negative band, typical of the α-helix at around 222 nm sums to the negative band due to positive couplet. The final result is that the minimum at around 220 nm is more intense than the minimum at 207 nm.

#### Differential Scanning Calorimetry

**Figure 4:**
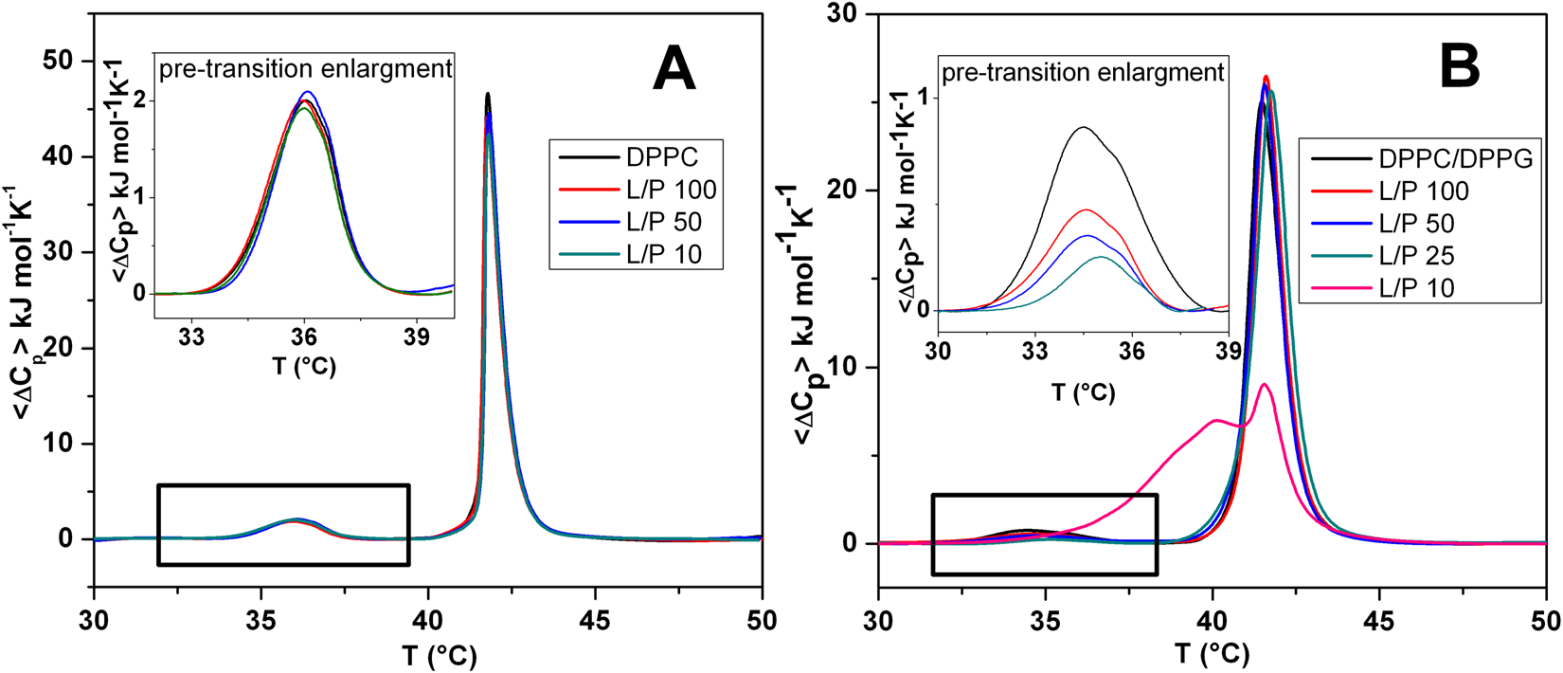
DSC profiles of DPPC (**A**) and DPPC/DPPG (**B**) MLVs at the indicated lipid-to-peptide ratios. The insets show an enlargement of the pre-transition peaks.

**Figure 5:**
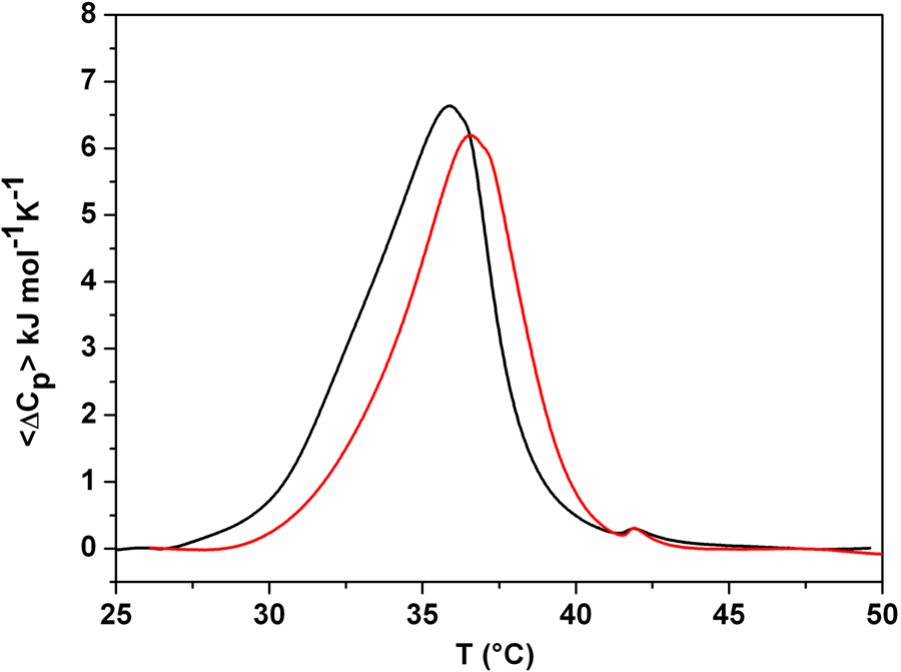
DSC profiles of DPPC/POPG (8/2 mol/mol) vesicles in the absence (black line) and the presence of P9Nal(SS) (red line) at lipid-to-peptide ratio of 50.

In order to study the effect of the peptide P9Nal(SS) on the lipid bilayers stability, differential scanning calorimetry (DSC) measurements were performed on the DPPC and DPPC/DPPG vesicles. The DSC curves of DPPC and DPPC/DPPG vesicles in the absence of peptide are very similar and characterized by two well-defined transitions. The transition from the lamellar gel phase to the rippled gel phase, named pre-transition (at about 36 °C), is due to a rearrangement of polar head groups; the second transition (main-transition), from the rippled gel phase to liquid phase (at 41.8 °C), is due to the melting of hydrocarbon chains of lipids (Fig. 4)^49–51^. The changes induced by the presence of the peptide on the thermotropic properties of these transitions offer the opportunity to investigate the effect of the peptide on two distinct regions of lipid bilayer. Figure 4A and B report the DSC thermograms of DPPC and DPPC/DPPG vesicles on increasing P9Nal(SS) concentration. P9Nal(SS) has only a small effect on the DPPC thermotropic properties (Fig. 4A), suggesting that the peptide weakly interacts with the DPPC bilayer at the surface level and does not significantly perturb the lipid organization in the membrane. The peptide shows a strong effect for the DPPC/DPPG bilayer (Fig. 4B), as indicated by the modifications of both the pre-transition and the mean transition peaks. Inspection of the thermograms reveals that P9Nal(SS) is able to induce a modification of the pre-transition peak even at low peptide concentration (high L/P ratios). In particular, the enthalpy change decreases and the peak is shifted at higher temperature as the peptide concentration increases, (inset of the Fig. 4B). A slightly different result was obtained for the main-transition: the presence of peptide does not affect the main phase transition until L/P = 50, at L/P = 25 a slight modification of the peak was observed, and at L/P = 10 a dramatic change occurred. These observations suggest that the interaction of the peptide with the DPPC/DPPG bilayer strongly depends on peptide concentration. In fact, at low peptide concentration, the interaction takes place only at the surface (affecting the pre-transition but not the main transition). At higher concentration (low L/P ratios), the peptide is able to penetrate into the bilayer hydrophobic core disrupting the regular packing of lipid acyl chains, as assessed by the dramatic modification of the main transition peak at L/P = 10 ratio. Further, the peptide is able to perturb the lipid distribution inducing the formation of domains of different lipidic compositions as demonstrated by the presence of a multicomponent DSC profile (Fig. 4B). Most likely, the domain formation is triggered by a P9Nal(SS) preferential binding to the negatively charged DPPG lipid. Unfortunately, a preferential interaction for the anionic lipid cannot be unambiguously demonstrated using DPPC/DPPG liposomes, as pure DPPC and DPPG bilayers have very similar main transition temperatures. To verify the ability of P9Nal(SS) to induce lipid segregation, we repeated the DSC experiments by replacing DPPG lipid with POPG in the mixed vesicles. This replacement has several advantages: a) DPPC and POPG lipids are completely miscible at the POPG mole fraction used in this experiment^52^; b) POPG vesicles have a transition temperature below 0 °C and, thus, can affect the observed DPPC/POPG transition only indirectly by changing his local distribution. The DSC peak of the DPPC/POPG (8/2 mol/mol) mixture shows a value of the temperature for the gel-to-liquid phase transition centered at 35.9 °C (Fig. 5). In the presence of P9Nal(SS), at the L/P = 50, the transition temperature shifts at 36.5 °C and the peak appears sharper than in the absence (i.e. the transition is more cooperative). This result strongly support the evidence that the peptide prefers to interact with POPG molecules inducing a lipid segregation that bring to the formation of DPPC-rich domains characterized by a higher transition temperature (Fig. 5).

#### Fluorescence Anisotropy

**Figure 6:**
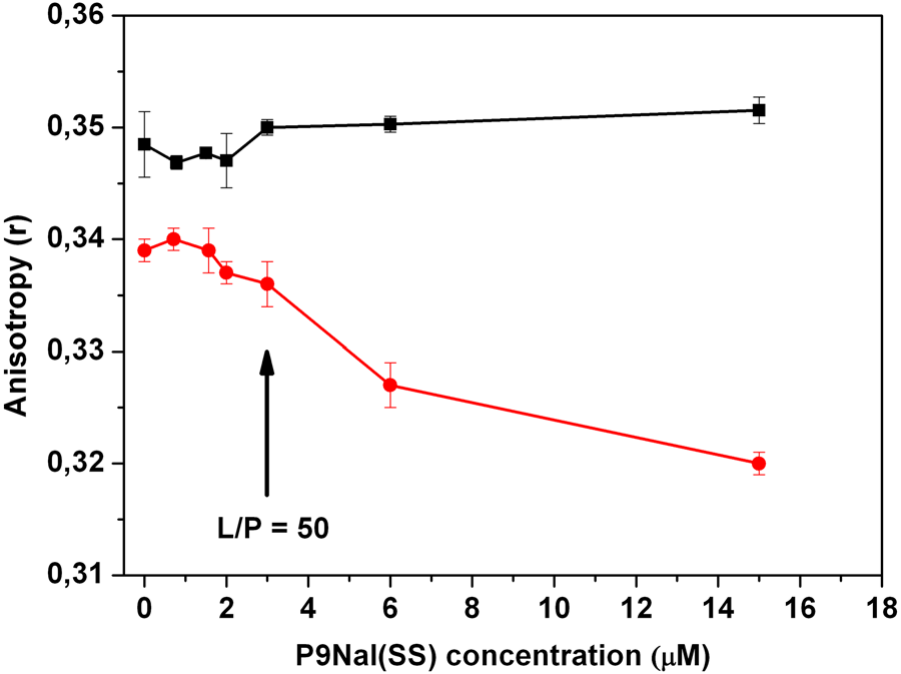
Fluorescence anisotropy of DPH embedded in DPPC (black squares) and DPPC/DPPG (red circles) vesicles as function of P9Nal(SS) concentration.

To get further insight into the effect of peptide on the stability of lipid bilayer, fluorescence anisotropy measurements were performed on the vesicles labeled with DPH^53,54^. Measuring DPH anisotropy values can give information about the micro-viscosity of the membrane (that in turn, depends on lipid acyl chains packing) and the effect of the peptide on it. Figure 6 shows the values of anisotropy of DPH embedded either in DPPC or DPPC/DPPG vesicles, as function of peptide concentration. P9Nal(SS) doesn’t affect the anisotropy values of DPH embedded in DPPC vesicles (the value remains constant at about 0.35), thus suggesting that no penetration into the bilayer occurs according to DSC results. For the DPPC/DPPG system, the anisotropy values of DPH remains roughly constant until L/P = 50 and decreases at higher peptide concentration (L/P < 50). These results confirm that P9Nal(SS) is able to penetrate inside the hydrophobic core with a concentration-dependent mechanism.

#### Fluorescence Resonance Energy Transfer

**Figure 7:**
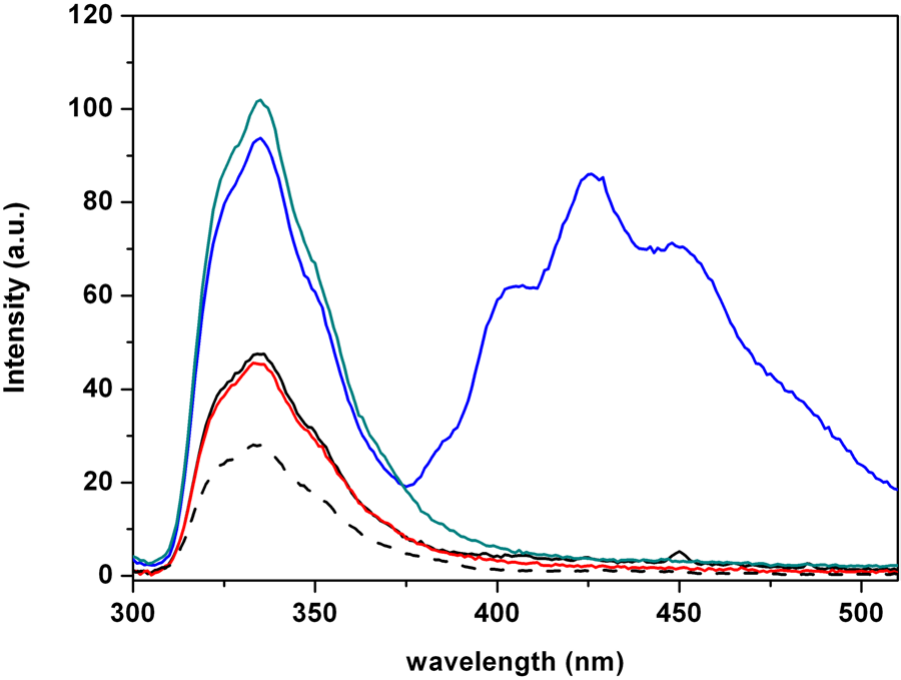
Fluorescence emission spectra of P9Nal(SS) and DPH with DPPC (black line) and DPPC/DPPG (blue line) vesicles at fixed L/P = 50. As references, the emission spectra of P9Nal(SS) in phosphate buffer in the absence of lipid vesicles (black dashed line) and in the presence of DPPC (red line) and DPPC/DPPG (green line) without DPH are reported.

As the fluorescence emission spectrum of the peptide is superimposable to the absorption spectrum of DPH embedded in the lipid vesicles, the peptide and DPH can be used as donor-acceptor pair in a FRET experiment, to analyze peptide vesicles interactions^54^. FRET experiment was carried out exciting the peptide at λ_ex_ = 277 nm and recording the fluorescence emission up to 510 nm, including the region of emission of DPH. Figure 7 shows the emission spectra of peptide and DPH when embedded in DPPC (red line) and in DPPC/DPPG (blue line) vesicles. The fluorescence spectra of the peptide in the presence of the vesicles without DPH have been also reported as references. The intensity emission of P9Nal(SS), centered at 337 nm, increases in the presence of both lipid vesicles in the absence of DPH, confirming that the peptide binds both DPPC and DPPC/DPPG bilayers. Interestingly, when DPH was added to the vesicles, FRET emission in the range 400–510 nm and the corresponding fluorescence decrease at 340 nm were observed only for the DPPC/DPPG vesicles.

These results can be interpreted considering the main factors affecting FRET^39^. The occurrence of FRET between a donor (D) and an acceptor (A) mainly depends on three parameters: 1) the overlap integral, J(λ), i.e. superposition of the emission spectrum of D and absorption spectrum of A; 2) the distance between D and A and 3) the relative orientation (κ^2^) between them. For the bilayers analyzed, 1) and 2) should be very similar. Therefore, the observed difference in FRET intensity could be mainly attributed to differences in the relative orientation between the fluorophores of the peptide and DPH. The lack of the FRET in the case of DPPC suggests that in this case the orientation factor (κ^2^) is close to zero, indicating that the 2-naphthyl-L-alanine (2-Nal) residues on the peptide are positioned perpendicular to the DPH. This result is consistent with a binding of the peptide at the membrane surface, resulting in the 2-Nal residues positioned parallel to the vesicle surface and perpendicular to the DPH molecule. For the DPPC/DPPG vesicles the orientation factor (κ^2^) is not zero, consistent with a peptide insertion inside the bilayer.

#### Fluorescence Quenching

**Figure 8:**
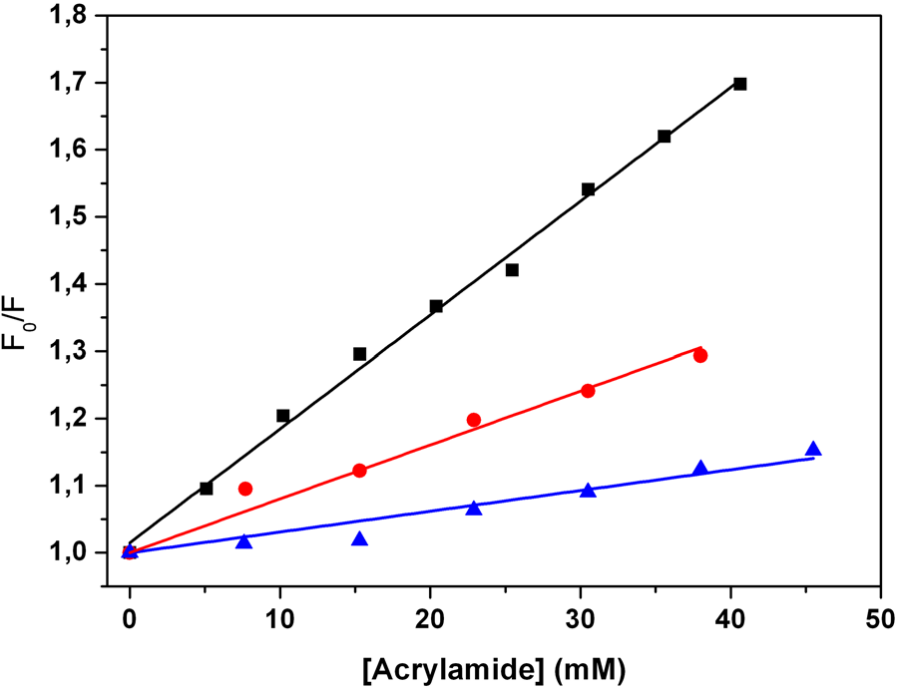
Stern-Volmer plots for the fluorescence quenching of P9Nal(SS) with a solution of acrylamide in buffer (black squares), in the presence of DPPC (red circles) and in the presence of DPPC/DPPG (blue triangles) SUVs at L/P of 100.

To get further insight on the degree of insertion of the peptide inside the lipid bilayer, fluorescence quenching experiment were performed. In particular, fluorescence emission spectra of the peptide in the absence and presence of DPPC or DPPC/DPPG vesicles, at fixed L/P = 100, were recorded at different acrylamide concentration (spectra not shown). A plot of F_0_/F versus [Q] gives a straight line whose slope represents the Stern-Volmer constant (K_SV_), accordingly to Equation 2 (see Methods Section). The value of K_SV_ is an index of the degree of exposure to the aqueous solvent of the two 2-naphthyl-L-alanine (2-Nal) residues in the peptide sequence. The Stern-Volmer plots for the acrylamide fluorescence quenching of P9Nal(SS) in the absence and presence of DPPC and DPPC/DPPG vesicles are reported in Fig. 8. The greatest K_SV_ value of (16.9 ± 0.4) M^−1^ was found for P9Nal(SS) in buffer solution, because of the complete exposure of 2-Nal residues to the solvent. In the presence of DPPC vesicles, a value of K_SV_ of (8.0 ± 0.5) M^−1^ was obtained suggesting a reduced exposure to the solvent. In the presence of DPPC/DPPG vesicles, the K_SV_ value further decreases at (3.1 ± 0.4) M^−1^ suggesting that the peptide is inserted inside the membrane. These results are in agreement with FRET data, and support the hypothesis of a different mechanism for the interactions of the peptide with the two membranes.

#### Electron Paramagnetic Resonance Measurements

Spin-labeled substances (peptides and/or lipids) has been proved to give substantial information on the interaction between peptides/proteins and lipid membranes^55^. A well-assessed approach reported in the literature for membrane-interacting viral peptides was followed^41^.

**Figure 9:**
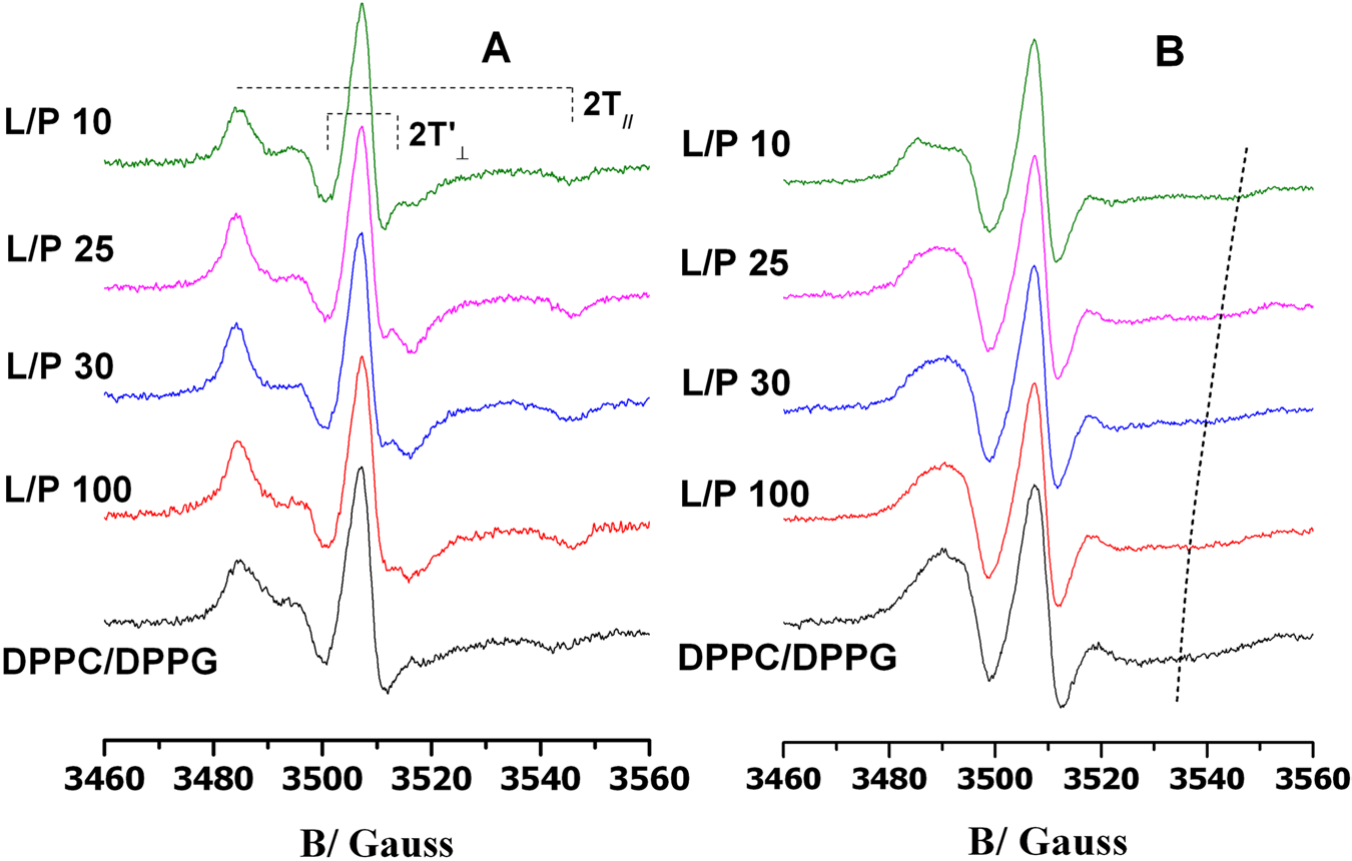
EPR spectra of 5-PCSL (**A**) and 14-PCSL (**B**) of the DPPC/DPPG 80/20 (mol/mol) lipid bilayer, in absence of peptide (black curve) and in presence of P9Nal(SS) at different lipid to peptide molar ratio (dyed curves). The dashed line highlights the high-field minimum in the 14-PCSL spectra.

**Figure 10:**
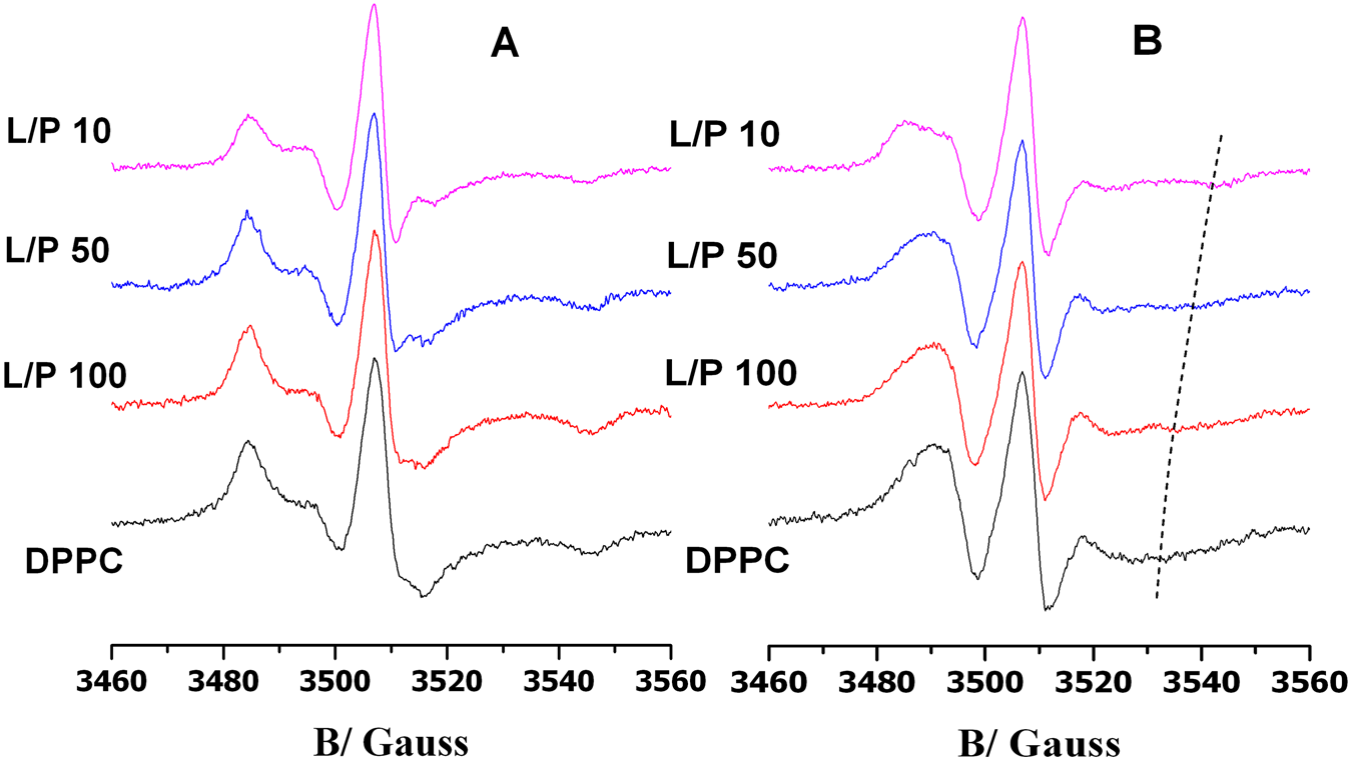
EPR spectra of 5-PCSL (**A**) and 14-PCSL (**B**) of the DPPC lipid bilayer, in absence of peptide (black curve) and in presence of P9Nal(SS) at different lipid to peptide molar ratios (dyed curves). The dashed line highlights the high-field minimum in the 14-PCSL spectra.

Perturbation caused by P9Nal(SS) binding to DPPC and DPPC/DPPG bilayers was investigated by analyzing changes in the EPR spectra of spin-labeled lipids included in the membrane. The 5-PCSL and 14-PCSL spectra in the two lipid systems, in the absence and in presence of P9Nal(SS) at a different lipid to peptide molar ratio, are shown in Figs 9 and 10 for DPPC/DPPG and DPPC vesicles, respectively. 5-PCSL presents the nitroxide reporter group close to the hydrophilic lipid head-group, while in 14-PCSL, the nitroxide group is positioned close to the terminal methyl group. Thus, a combined use of the two spin-labels permits the monitoring of both the outer membrane regions and its inner core, since the analysis of the spectra furnishes information about the local microstructure and polarity. In order to quantitatively analyze the spectra, we evaluated the order parameter, S, and the isotropic hyperfine coupling constant, a′_N_, using a spectrum parametrization method described in the literature^41^. S is a measure of the local orientational ordering of the labeled segment of the acyl chain with respect to the normal to the bilayer surface. a′_N_ is an index of the micropolarity experienced by the nitroxide^56^. Inspection of Fig. 9 shows that addition of P9Nal(SS) to DPPC/DPPG liposomes causes an increases of the 5-PCSL spectrum, as highlighted by the shift of the high-field minimum and the enhanced resolution of the low-field maxima. With increasing the peptide content, the spectrum does not show further changes, until a L/P = 10 ratio is reached, at which a reduction of the anisotropy is detected. On the other hand, the 14-PCSL spectrum in DPPC/DPPG in absence of the peptide presents a broad three-lines shape. P9Nal(SS) scarcely perturbs the spectrum; only at the lowest considered L/P ratio the peptide causes an evident splitting of the low-field maximum and the appearance of an additional high-field minimum.

The values of the order parameter, S, and isotropic hyperfine coupling, a′_N_, for the DPPC/DPPG lipid system, collected in Table 3, quantitatively confirm that P9Nal(SS) induces a significant increment of the lipid ordering close to the bilayer interface, with the exception of the values obtained at L/P = 10 which is lower. At this ratio, an increment of the inner core local structuring is clearly observed. These results support a change in the peptide/membrane interaction mechanism, from a surface adsorption (at high L/P ratio) to a deeper penetration among the lipid acyl chains (at low ratio). Interestingly, At L/P = 10 polarity in the membrane interior significantly increases (as highlighted by the higher a′_N_ value), suggesting that inserted peptides maintain, at least partially, solvating water molecules.

**Table 3:** Order parameter, S, and hyperfine coupling constant, a′_N_, of *n*-PCSL in DPPC and DPPC/DPPG liposomes, in the absence and presence of P9Nal(SS)^a^.

		5-PCSL		14-PCSL	
		S	a′_N_/G	S	a′_N_/G
DPPC		0.87	15.2	0.43	14.7
	+P9Nal(SS) (L/P = 100)	0.87	15.2	0.48	14.6
	+P9Nal(SS) (L/P = 50)	0.86	15.2	0.49	14.8
	+P9Nal(SS) (L/P = 30)	0.79	15.6	0.60	16.8
DPPC/DPPG		0.73	15.9	0.46	15.0
	+P9Nal(SS) (L/P = 100)	0.86	15.3	0.51	15.1
	+P9Nal(SS) (L/P = 30)	0.89	15.1	0.53	15.0
	+P9Nal(SS) (L/P = 25)	0.90	15.1	0.55	15.2
	+P9Nal(SS) (L/P = 10)	0.84	15.4	0.65	16.8

^a^Estimated error in S is <±2%; in a′_N_, 0.2 G.

5-PCSL and 14-PCSL spectra in DPPC bilayers are reported in Fig. 10; it is to be noted that this lipid systems forms bilayers whose interface is more ordered with respect to DPPC/DPPG bilayers, as also highlighted by the S values reported in Table 3. The presence of P9Nal(SS) causes a slight reduction of the 5-PCSL spectrum anisotropy, which becomes more evident at low L/P values, as also detectable by the decrease of the S value. The 14-PCSL spectrum shows a progressive anisotropy increase with decreasing the L/P ratio, which reflects in an increase of the S values. The a′_N_ value shows a certain increase of the local polarity in the bilayer core, which however never reach that observed for the DPPC/DPPG bilayer. Thus, the data suggest that P9Nal(SS) interaction with DPPC bilayers is different from that observed with DPPC/DPPG membranes. For charged bilayers an initial absorption at the bilayer interface is clearly detected, followed by a deep insertion at lowest lipid to peptide ratio. When zwitterionic membranes are considered, the mechanism of interaction is less defined. Nevertheless, in both cases the peptide considerably affects the lipid self-organization, which could finally lead to the membrane destabilization and loss of functionality.

## Discussion

Antimicrobial peptides are promising candidates as future therapeutics in order to face the problem of antibiotic resistance caused by pathogenic bacteria. A key issue to be addressed for the development of synthetic peptides as antimicrobial agents is to develop sequences that display both resistance to proteolysis and selective toxicity. Indeed, the therapeutic applications of CAMPs are limited by their sensitivity to proteases, which drastically reduces their half-life, and/or for their concomitant toxic activity. Several researches have developed CAMPs with modifications that reduce sensitivity to proteases, like blocking N- and C-termini, introducing D-amino acids at key positions, etc.^29,30^. The three peptides used in this work were designed to mimic naturally occurring CAMPs but un-natural amino acids were included in their sequences to increase their stability toward bacterial and host (e.g. serum) proteases, in order to obtain pharmacologically improved compounds with increased activity and prolonged half-life *in vivo*. The results of the serum stability assay clearly showed that the three designed peptides are more stable than “natural” CAMPs, in addition they also revealed intriguing differences among the three synthetic peptides, in fact, the designed peptide P9Nal(SR) completely lost its activity after 16 h of incubation in serum, whereas, P9Nal(SS) and P9Trp(SS) were still active even if showing a four fold and eight fold increase in the MIC values, respectively. These results suggest that 2-Nal and S-(S-tert-butylthio) L-cysteine confer higher resistance to proteolysis than tryptophan and S-(tert-butylthio) L-cysteine, respectively. The two S-(S-tert-butylthio) L-cysteine residues seem to be particularly important given the relevant difference between the stabilities of P9Nal(SS) and P9Nal(SR). On the other hand it is well known that several other factors, such as cationic character and hydrophobicity, modulate both antimicrobial potency of CAMPs as well as their cell selectivity, and hence their toxicity^31^. To this end, we also evaluated the relative hydrophobicities of P9 peptides through their retention in reverse phase HPLC (Fig. S4 and Table S1). Analysis of the data revealed that peptide P9-Nal(SS) is the most hydrophobic, whereas the other two analogues show comparable hydrophobicity. Both results well correlate with the observed higher activity of P9-Nal(SS) against Gram-positive bacteria (Table 1). Indeed, it is well known that *S. aureus* strains secrete several proteases (e.g. aureolysin, staphopain A and staphopain B) involved in pathogenesis and resistance to host defense protein/peptides including complement factors and CAMPs^46^. Moreover, previous studies^57^ have demonstrated that an increase in hydrophobicity increases potency when Gram-positive bacteria are targeted. In contrast, such modification has limited effect when targeting Gram-negative species^58^. Therefore, the higher antimicrobial activity displayed by P9Nal(SS) could be partly related to its lower sensitivity to proteases and partly to its higher hydrophobicity. The cell toxicity assay showed that the three designed peptides exert significant dose dependent toxic effects on two different human cell lines (Fig. 2). P9Nal(SS) showed the highest toxicity whereas P9Nal(SR) showed a lower toxicity than the remaining two peptides, especially on the non transformed cell line HaCaT. Also in this case the high toxicity of P9Nal(SS) is likely related to its higher hydrophobicity and resistance to proteases. The fact that P9Nal(SR) is less toxic than P9Trp(SS), in spite of the similar hydrophobicity, could be the result of a faster degradation when incubated with cells due to the lower stability in serum of P9Nal(SR).

The biological characterization allowed to conclude that, even if our approach has been partially successful providing more stable and more active CAMPs, at the same time these CAMPs are endowed with a non selective toxicity toward prokaryotic and eukaryotic cells, hence they are not suited for a pharmacological use. However, it remains open the possibility to slightly modified the designed peptides to improve their selectivity without affecting their toxicity toward the prokaryotic cells. On this regard, there are several examples were other toxic non-cell selective CAMPs were converted into cell selective AMPs^59–62^. A single aminoacidic mutations has been shown to drastically reduce the hemolytic activity of melittin^59^. On the other hand, the incorporation of D-amino acids into antimicrobial peptide sequences had often resulted in disruption of hemolytic or cytotoxic properties of lytic peptides without lowering their antimicrobial activities^60,63–66^. Recently, it has been shown that the introduction of cationic residue(s) at the interface of polar and non-polar faces of piscidins may control its insertion into hydrophobic mammalian cell membrane and thereby reducing its cytotoxicity^62^. All these examples suggest that, in principle, a toxic non-cell selective peptide could be a good starting point for a further design aimed at decreasing the undesired toxicity. To more rationally plan possible peptide modifications, in the present study, we collected more detailed information on the peptide-membrane interaction mechanism. To this aim, we chose P9Nal(SS) for further biophysical studies as this peptide, among the three peptides, possesses broader spectrum antibacterial activity together with the higher stability in serum. Particularly, we characterized the interaction of P9Nal(SS) with DPPC and DPPC/DPPG liposomes mimicking the eukaryotic and bacterial membranes, respectively.

CD spectra showed that P9Nal(SS) conformation changes significantly when DPPC and DPPC/DPPG liposomes are added to the solution and seems to approach an helix-like conformation (Fig. 3). It is know that the membrane perturbation activity of many AMPs is related to their ability to adopt an helical conformation^67^. From this point of view, the ability of the peptide to adopt a similar conformation in the presence of both vesicles is consistent with the observed low selectivity. However, in contrast with this result, DSC experiments showed that the effects of P9Nal(SS) binding on the thermotropic properties of the membranes are strongly dependent on the membrane lipid composition. Particularly, P9Nal(SS) has only a small effect on the DPPC vesicles, suggesting that the peptide is not able to perturb the lipid packing and, thus, does not penetrate deeply in the bilayer hydrophobic core. On the contrary, DSC experiments revealed that P9Nal(SS) is able to drastically perturb the DPPC/DPPG vesicles in a concentration dependent manner. At low L/P ratio only the pre-transition is affected suggesting a binding of P9Nal(SS) at the lipid heads groups/water interface whereas at higher concentration the gel to liquid transition peak is drastically modified. This result strongly suggests that, at high concentration, the peptide is able to deeply penetrates into the bilayer hydrophobic core disrupting the regular packing of lipid acyl chains. Further, the peptide is able to perturb the lipid distribution inducing the formation of domains of different lipid compositions as demonstrated by the presence of a multicomponent DSC profile (Fig. 4B).

We speculated that the ability of P9Nal(SS) to perturb the DPPC/DPPG but not the DPPC vesicles could be attributed to a preferential interaction of the peptide with the negatively charged DPPG lipid that leads to lipid segregation and domain formation, as observed for other cationic AMPs^12,68,69^. To experimentally verify this hypothesis, we repeated the DSC experiment by replacing DPPG with POPG lipid in the mixed vesicles. The main advantage of this experiment is that POPG does not directly contribute to the observed transition and, thus, can affect the observed DPPC/POPG transition only indirectly by changing its local distribution. The obtained results support the hypothesis that the peptide prefers to interact with POPG molecules inducing a lipid segregation that bring to the formation of DPPC-rich domains that melts at higher temperature and more cooperatively. The formation of such lipid domains could be crucial for the peptide action as lipid domains can act as defects destabilizing the double layers^12,70,71^ and facilitating the peptide penetration into the membrane hydrophobic core.

The ability of P9Nal(SS) to penetrate in the DPPC/DPPG membrane but not in the DPPC membrane was further demonstrated by the fluorescence experiments with the DPH containing membranes. Monitoring the DPH anisotropy variation upon peptide addition is a powerful method to follow the peptide penetration into the membrane hydrophobic core. In these experiments we observed no anisotropy variation in the case of DPPC and a strong variation in the case of DPPC/DPPG. Interestingly, we observed that P9Nal(SS) affects the DPH anisotropy embedded in the DPPC/DPPG vesicle after a threshold peptide concentration in agreement with the DSC result. The presence of an L/P threshold concentration is common to several AMPs and it seems to be a necessary requisite for their biological activity^12^. Further, a threshold concentration is always required for membrane disruption almost independently of the mechanism of action being a pore-formation mechanism or a carpet-like mechanism^72–74^.

In summary, DSC and fluorescence anisotropy measurements are consistent with a concentration dependent membrane perturbation mechanism of P9Nal(SS) with respect the DPPC/DPPG vesicles. Particularly, the peptide binds to the membrane surface inducing anionic lipid segregations and above a threshold P/L value the peptide penetrates deeply into the hydrophobic core of the membrane. On the contrary, all the data show mainly surface binding of P9Nal(SS) on the DPPC bilayer even at low L/P ratio and a not significant membrane perturbation.

All these findings, together with the biological results, suggest that the mechanisms responsible for the antimicrobial activity could be different to that responsible for the concomitant undesired cytotoxicity. These results could represent an important contribution for future development of new peptides as antimicrobial agents.

## Electronic supplementary material

Supplementary Information
