# Supplementary material for: Exploring the role of unnatural amino acids in antimicrobial peptides
Source: Sci Rep. 2018 Jun 11;8:8888. doi: 10.1038/s41598-018-27231-5 (PMC5995839; doi:10.1038/s41598-018-27231-5)
Supplement: Supplementary file 1 — Supplementary Information [file 41598_2018_27231_MOESM1_ESM.pdf]

Supplementary Material

Exploring the role of unnatural amino acids in antimicrobial peptides

Rosario Oliva[1], Marco Chino[1], Katia Pane[2], Valeria Pistorio[3], Augusta De Santis[1], Elio Pizzo[2], Gerardino D'Errico[1], Vincenzo Pavone[1], Angela Lombardi[1], Pompea Del Vecchio[1], Eugenio Notomista[2], Flavia Nastri[1], Luigi Petraccone[1*]

[1]Department of Chemical Sciences, University of Naples "Federico II", via Cintia, I-80126 Naples, Italy

[2]Department of Biology, University of Naples "Federico II", Via Cintia, I-80126 Naples, Italy

[3] Department of Molecular Medicine and Medical Biotechnologies, University of Naples "Federico II", Via Pansini, 5, I-80131 Naples, Italy

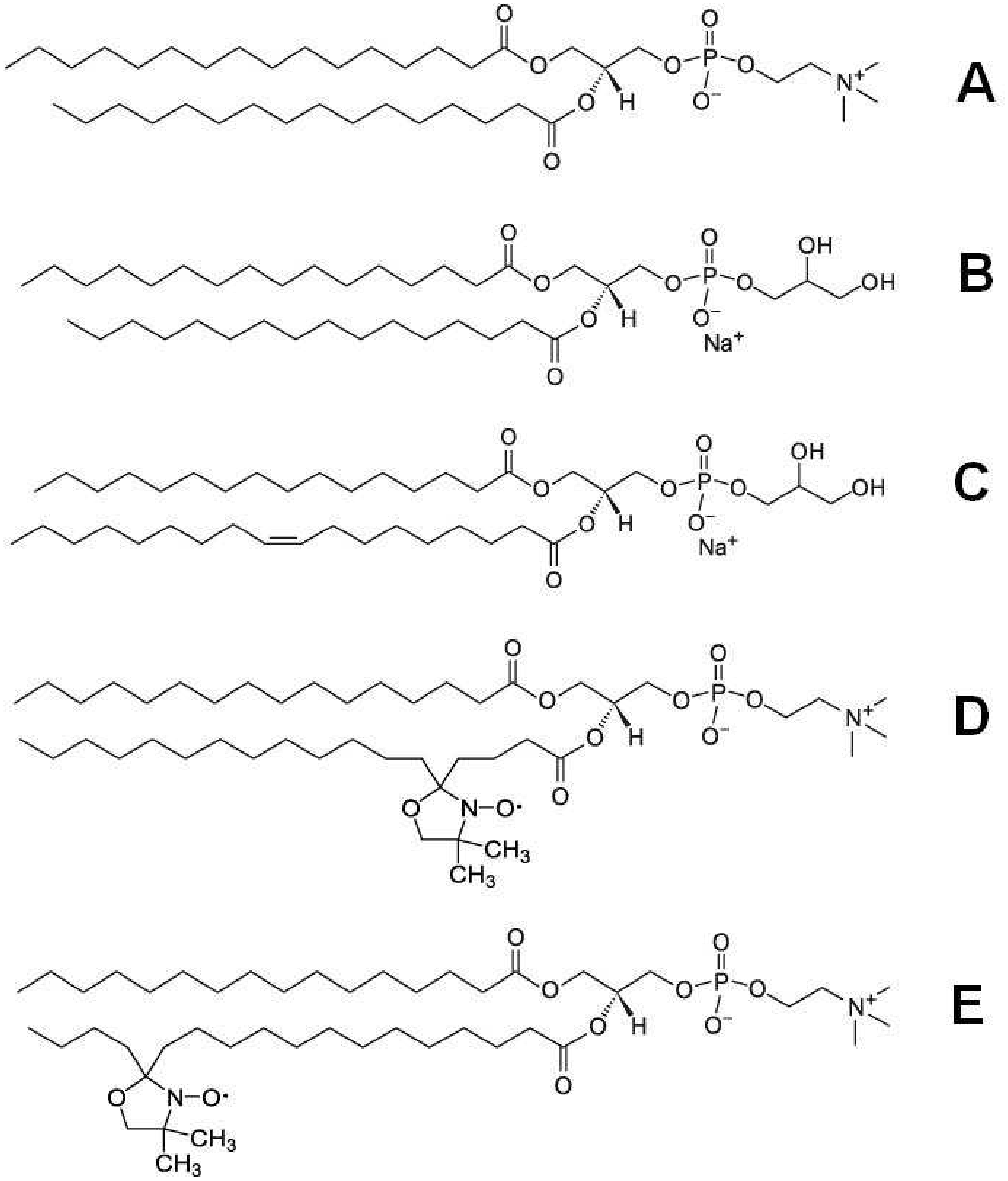

Fig. S1 The chemical structure of lipids used in this study: (A) DPPC, (B) DPPG, (C) POPG, (D) 5-PCSL and (E) 14-PCSL.

The accurate determination of peptide concentration is essential for quantitative biophysical, molecular studies. We determined the molar extinction coefficient of P9Nal(SS) peptide by spectrophotometric method. The absorbance of ultraviolet (UV) radiation at 277 nm was measured at different peptide concentrations. Peptide samples at fixed concentration were prepared by weighting the peptide as lyophilized dry powder. The molar extinction coefficient, ε = (9109 ± 73) M^{-1} cm^{-1} was calculated by the Lambert-Beer slope plot. The cell length was 0.1 cm and the temperature 25°C.

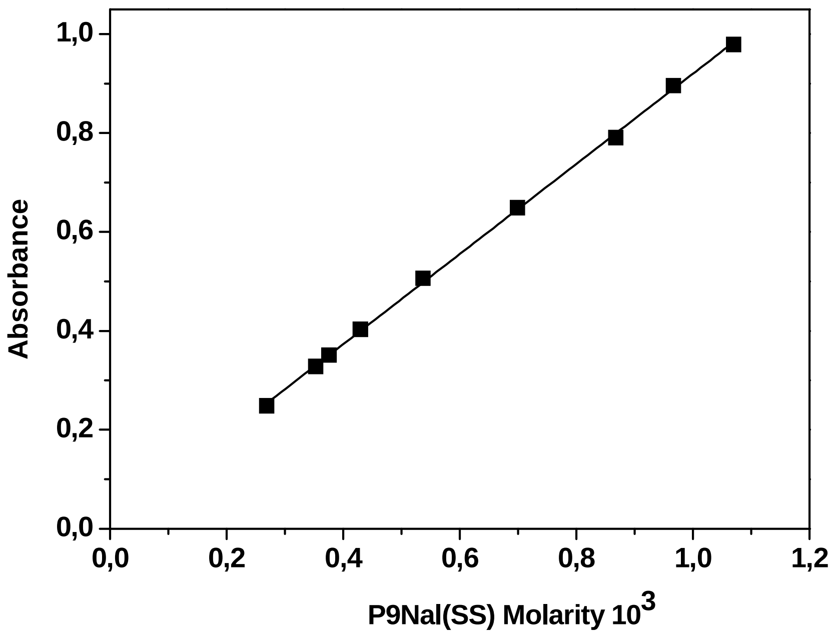

Fig. S2: Plot of the absorbance at 277 nm versus P9Nal(SS) concentration. The solid line is the best line fit obtained using the Lambert-Beer equation.

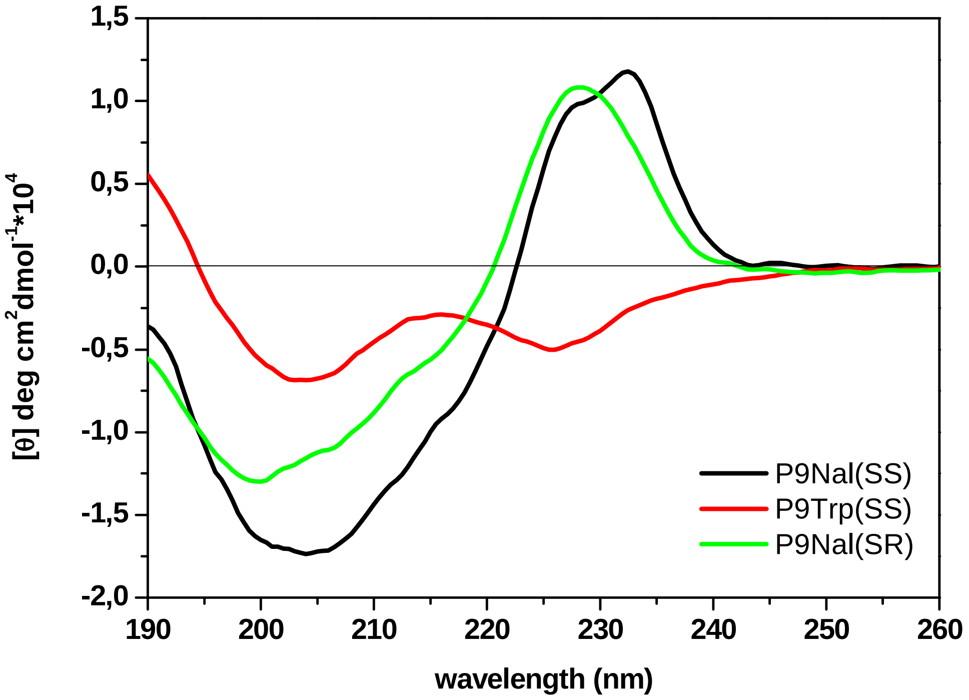

Fig. S3 : Far-UV CD spectra of P9Nal(SS) (black line), P9Trp(SS) (red line) and P9Nal(SR) (green line) peptides recorded in 20 mM phosphate buffer, pH 7.4 at temperature of 25 °C.

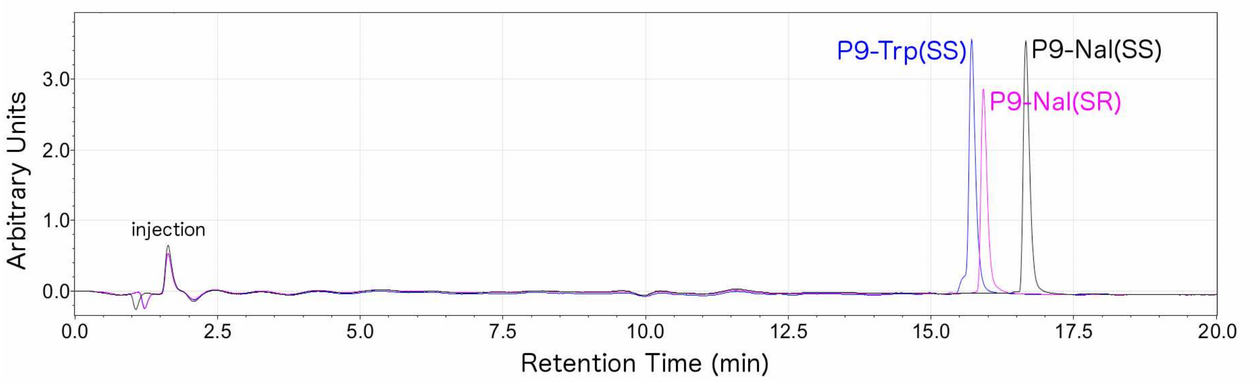

Fig. S4 Analytical RP-HPLC chromatograms collected at 280 nm upon injections of P9-Nal(SS) (black line), P9-Nal(SR) (pink line) and P9-Trp(SS) (blue line).

Table S1 Relative hydrophobicity of P9 analogues.*

Peptide	retention time (min)	% B
**P9-Nal(SS)**	16.7	57.4
**P9-Nal(SR)**	15.9	54.1
**P9-Trp(SS)**	15.4	53.2

* Expressed as percentage of acetonitrile (% B) at peak elution on analytical RP-HPLC
